# Sentiment and Thematic Analysis of User Reviews for FDA-Cleared Prescription Digital Therapeutics: A Mixed-Methods Real-World Evidence Study

**DOI:** 10.7759/cureus.84710

**Published:** 2025-05-23

**Authors:** Shaheen E Lakhan

**Affiliations:** 1 Medical Office, Click Therapeutics, Inc., New York, USA; 2 Department of Neurology, Western University of Health Sciences, Pomona, USA; 3 Department of Neurology, A.T. Still University School of Osteopathic Medicine in Arizona, Mesa, USA; 4 Department of Neurology, Morehouse School of Medicine, Atlanta, USA; 5 Department of Bioscience, Global Neuroscience Initiative Foundation, Miami, USA

**Keywords:** app store reviews, digital therapeutics, fda-cleared software, mobile health, patient experience, pdt, prescription digital therapeutics, real-world evidence, sentiment analysis, thematic analysis

## Abstract

Background

Prescription digital therapeutics (PDTs) are FDA-cleared, evidence-backed, smartphone-based interventions. While clinical trials establish efficacy under controlled conditions, the patient experience in real-world settings remains poorly characterized. App store reviews, though informal, offer a potential source of real-world evidence (RWE) reflecting user sentiment, barriers to engagement, and perceived benefit.

Objective

To characterize the real-world user experience of FDA-cleared PDTs through a mixed-methods analysis of publicly available app store reviews, combining quantitative sentiment classification with qualitative thematic analysis.

Methods

FDA-cleared PDTs via FDA’s De Novo or 510(k) pathways were identified. Reviews were collected from the Apple App Store and Google Play Store using structured scraping tools. A rules-based sentiment classifier was applied based on user star ratings, and thematic content analysis was conducted using a hybrid natural language processing and manual coding approach. Reviews were grouped by app and platform, and dominant themes were extracted using unsupervised topic modeling.

Results

Of the 13 PDTs identified, seven had publicly accessible user reviews, yielding a dataset of 247 unique entries: AspyreRx, EndeavorRx, Regulora, Rejoyn, reSET, reSET-O, and Stanza. Sentiment classification revealed that 25.1% of reviews were positive, 12.6% neutral, and 62.3% negative. Thematic analysis identified six recurrent themes, including pediatric benefit and engagement, rewards and incentives, access and activation barriers, technical issues, emotional reactions and cost sensitivity, and boredom and frustration. While negative sentiment was more prevalent overall, several products, particularly Rejoyn and EndeavorRx, received meaningful reports of perceived benefit and therapeutic value. Company transitions were noted to have disrupted the continuity of historical review data of some PDTs.

Conclusions

App store reviews provide a rich, user-centered source of RWE and insights for PDTs. They highlight dimensions of access, usability, and trust not typically captured in clinical trials. Integrating such feedback into post-market evaluation may inform future design, regulation, and reimbursement of software-based therapeutics.

## Introduction

Prescription digital therapeutics (PDTs) represent a rapidly expanding category of evidence-backed, smartphone-based interventions authorized by the US Food and Drug Administration (FDA) to treat a range of medical and psychiatric conditions [1]. Delivered via smartphones and other connected devices, these therapeutics offer scalable, non-pharmacologic alternatives or adjuncts to traditional care. Unlike wellness apps or consumer-grade mental health tools, PDTs undergo regulatory review for safety and effectiveness and are typically prescribed by clinicians. As such, they occupy a unique intersection between regulated medicine and user-directed technology.

While randomized controlled trials (RCTs) remain the gold standard for demonstrating clinical efficacy, they often overlook the real-world complexities of therapeutic engagement. In the context of PDTs, factors such as usability, accessibility, onboarding friction, and digital literacy may significantly influence adoption and adherence. Yet, these dimensions are rarely captured in formal trial endpoints. With PDTs increasingly reaching patients through app marketplaces, the post-market landscape is shaped not only by clinical outcomes but also by user experience.

Publicly available app store reviews offer a unique lens into this evolving dynamic. Though informal and unstructured, these reviews reflect the lived experiences of patients and caregivers navigating digital interventions in their daily lives. They highlight what is working, what is breaking down, and where gaps exist between regulatory intent and user reality. As such, they represent a novel but underutilized source of real-world evidence (RWE), one that may reveal barriers, facilitators, and unintended consequences not readily visible to regulators, developers, or clinicians.

This study leverages natural language processing (NLP) and qualitative analysis to systematically examine user reviews of FDA-cleared PDTs. By extracting sentiment and identifying recurring themes, we aim to better understand how these therapies are perceived in practice and what implications such feedback holds for future design, deployment, and evaluation. This study uses a mixed-methods approach, combining quantitative sentiment classification with qualitative topic modeling to generate exploratory insights into patient-reported experiences. In doing so, we move beyond clinical endpoints to ask a more foundational question: What do patients really think of their digital prescriptions?

## Materials and methods

Study design and objectives

This retrospective, mixed-methods study aimed to evaluate user-reported experiences with FDA-cleared PDTs by analyzing publicly available app store reviews. The primary objective was to extract sentiment and thematic content to better understand how these therapeutics are perceived and experienced in real-world settings. As software-based interventions increasingly enter regulated therapeutic domains, user feedback offers critical insights into engagement, usability, and perceived effectiveness - dimensions not always captured in clinical trials.

Eligibility criteria

Candidate PDTs were identified through a structured review of the FDA's public databases for De Novo and 510(k) medical device clearances [2], supplemented by developer websites and public app store listings [3,4]. To be eligible for review analysis, each digital therapeutic was required to (1) have FDA clearance as a prescribed PDT for the treatment of a diagnosed condition, (2) be publicly available on either the Apple App Store or Google Play Store, (3) support English-language use, and (4) have a minimum of one publicly posted user review in English. Apps were excluded if they were not yet commercially launched, had been withdrawn from the app store at the time of data collection, or were only accessible via closed-loop channels such as provider-issued activation codes. Products were not excluded based on therapeutic area, patient population, or sponsoring organization.

Data collection procedures

Data were collected on May 3, 2025, using Apify, a browser-based automation platform that enables programmatic scraping of web content [5]. For Android applications, the “Google Play Reviews Scraper” actor was configured to extract up to 1,000 English-language reviews per app from the US storefront, sorted by recency. For iOS applications, the “Apple App Store Review Scraper” actor was similarly configured to retrieve recent English-language reviews. Review-level metadata included star rating (on a 1-5 scale), review title, full body text, submission date, and platform source (Apple or Google). Non-English, promotional, or clearly autogenerated reviews were excluded, and no user identifiers were collected. All data were stored in structured CSV format for downstream analysis.

Sentiment classification

Sentiment classification was conducted using a rules-based approach anchored to the star ratings assigned by users in app store reviews. Reviews rated 4 or 5 stars were classified as positive, those rated 3 stars as neutral, and reviews with 1 or 2 stars as negative. This approach was chosen for its transparency and scalability across platforms. Reviews without a valid star rating were excluded from sentiment analysis. Data processing and classification were conducted using Microsoft Excel (Microsoft Corporation, Redmond, WA), and all results were exported to CSV format to ensure reproducibility and auditability. While this method allowed efficient classification across a large dataset, it may overlook cases where review text contradicts the star rating or includes sarcasm, ambiguity, or mixed sentiment. Given small subgroup sizes, no inferential statistics were performed, and all results are reported descriptively as counts and percentages. While rules-based classification ensured transparency, we acknowledge this method may not capture cases where the narrative tone contradicts the numerical rating. Reviews with ambiguous or missing ratings were excluded from sentiment analysis.

Thematic analysis

Thematic analysis was conducted using a hybrid computational and manual approach to identify recurring patterns in user experience across app store reviews. Text preprocessing included lowercasing, stopword removal, and tokenization using Python libraries, including NLTK (Natural Language Toolkit, University of Pennsylvania, Philadelphia, PA) and scikit-learn (Inria, Paris, France). Data structuring and manipulation were performed using Pandas (Python Software Foundation, Wilmington, DE). Unsupervised topic modeling was performed using non-negative matrix factorization as implemented in scikit-learn, a dimensionality-reduction technique that groups textual data based on shared vocabulary features [6,7]. This method was chosen for its interpretability and suitability for short-text clustering. Manual review was conducted by a single coder using an iterative approach to identify clinically meaningful themes. While inter-rater reliability was not calculated, consistency was maintained through repeated cross-checking of representative excerpts.

Each topic was reviewed and labeled manually based on its most frequent keywords and representative user reviews. Themes were assigned through an iterative process to ensure coherence and clinical relevance. Representative quotes were selected for each final theme to provide qualitative depth and context. Given small subgroup sizes, no inferential statistics were performed, and all results are reported descriptively as counts and percentages.

Ethical considerations

All data were derived from publicly accessible, anonymous user reviews posted in the Apple App Store and Google Play Store. No user contact occurred, and no protected health information was accessed. All quoted user content was paraphrased to preserve anonymity and prevent reidentification. Product names were included due to their public FDA clearance status and to preserve context for real-world interpretation. The study was deemed exempt from institutional review board oversight under prevailing guidelines for secondary use of public data.

## Results

Included therapeutics

A total of 13 FDA-cleared PDTs were initially screened for inclusion based on publicly available regulatory clearances and active listings in the Apple App Store and Google Play Store [2-4]. Five PDTs were excluded due to an absence of publicly accessible user reviews, likely reflecting restricted distribution or recent market entry: Somryst (Nox Health, Alpharetta, GA), MamaLift Plus (Curio Digital Therapeutics, Princeton, NJ), SleepioRx (Big Health, San Francisco, CA), DaylightRx (Big Health, San Francisco, CA), and CT-132 (Click Therapeutics, New York, NY). An additional product, Parallel (Mahana Therapeutics, San Francisco, CA), was excluded after being confirmed as withdrawn from commercial availability at the time of data collection.

The final analytic sample included seven PDTs that were actively listed in at least one app store with one or more English-language user-generated reviews: AspyreRx (Click Therapeutics, New York, NY), EndeavorRx (Akili Interactive, Boston, MA), Regulora (metaMe Health, Chicago, IL), Rejoyn (Otsuka Precision Health, Princeton, NJ), reSET (PursueCare, Middletown, CT), reSET-O (PursueCare, Middletown, CT), and Stanza (Swing Therapeutics, San Francisco, CA).

Sentiment analysis

Across the final dataset of 247 user reviews, sentiment was classified using a rules-based approach anchored to user-submitted star ratings. A total of 62 reviews (25.1%) were classified as positive, 31 (12.6%) as neutral, and 154 (62.3%) as negative. Sentiment distributions varied considerably across products. EndeavorRx, the only included PDT authorized for pediatric use, accounted for the largest volume of reviews (127 total), with a strong skew toward negative sentiment: 30 (23.6%) positive vs. 81 (63.8%) negative. In contrast, reSET-O for opioid use disorder had a more balanced distribution: 24 (27.3%) positive, 14 (15.9%) neutral, and 50 (56.8%) negative. Rejoyn, recently cleared for major depressive disorder, showed a polarized pattern with 7 (35.0%) positive and 12 (60.0%) negative reviews, with only 1 (5.0%) neutral. Products with very few reviews, such as Regulora, AspyreRx, Stanza, and reSET, had too few responses to infer consistent sentiment patterns. Table 1 summarizes the sentiment distribution across all included PDTs by count and proportion.

**Table 1:** Sentiment Classification of User Reviews for FDA-Cleared Prescription Digital Therapeutics Sentiment classification of app store reviews across seven FDA-cleared prescription digital therapeutics (PDTs), categorized as positive, neutral, or negative based on user-assigned star ratings. Reviews rated 4-5 stars were labeled as positive, 3 stars as neutral, and 1-2 stars as negative. Counts and percentages are provided for each sentiment category, along with total reviews per app. Data reflect combined reviews from the Apple App Store and Google Play Store. This is an author-generated table. ADHD, attention-deficit/hyperactivity disorder.

PDT (Indication)	Positive Sentiment (%)	Neutral Sentiment (%)	Negative Sentiment (%)	Total Reviews
EndeavorRx (ADHD)	30 (23.6%)	16 (12.6%)	81 (63.8%)	127
reSET-O (opioid use disorder)	24 (27.3%)	14 (15.9%)	50 (56.8%)	88
Rejoyn (major depressive disorder)	7 (35.0%)	1 (5.0%)	12 (60.0%)	20
reSET (substance use disorder)	0 (0.0%)	0 (0.0%)	6 (100.0%)	6
Stanza (fibromyalgia)	0 (0.0%)	0 (0.0%)	4 (100.0%)	4
AspyreRx (type 2 diabetes)	0 (0.0%)	0 (0.0%)	1 (100.0%)	1
Regulora (irritable bowel syndrome)	1 (100.0%)	0 (0.0%)	0 (0.0%)	1

Thematic analysis

Unsupervised topic modeling and manual coding yielded seven recurring themes that reflect the dimensions of user experience most frequently discussed in app store reviews. The most common was “Pediatric Benefit and Engagement” (n=108; 43.7%), encompassing both children’s gameplay experiences and caregiver-reported improvements in attention, focus, and daily functioning. This theme was predominantly associated with EndeavorRx; one caregiver noted, “My daughter actually looks forward to using it every day -- it’s the only thing that has helped her focus.” The second theme, “Rewards and Incentives” (n=32; 13.0%), captured reactions to gamified elements. While some users found these motivating, stating, “The rewards system made my son want to keep playing”, others questioned their substance, writing, “It’s all flashy prizes but no real progress.”

“Access and Activation Barriers” (n=20; 8.1%) emerged as another frequent concern, capturing user frustration with prescription access codes, clinician referrals, and waitlists. Comments such as “The app is telling me I need to upgrade but won’t let me” and “I’ve been on the waitlist for weeks with no update” were common. Technical Issues and Updates (n=18; 7.3%) included app crashes, failed updates, and compatibility problems, as noted in reviews like “Quit working after 3 weeks and now just crashes on load.”

The fifth theme, “Emotional Reactions and Cost Sensitivity” (n=15; 6.1%), featured polarized expressions such as “Worst app I’ve ever used -- waste of money,” along with skepticism about the value of digital therapeutics. “Boredom and Frustration” (n=9; 3.6%), though less common as the sixth theme, reflected disengagement due to repetitive or unstimulating content, as one user explained: “It got boring fast and didn’t feel like it was doing anything.” Finally, a number of reviews were categorized as “Unknown or Unclassified” (n=45; 18.2%) due to lacking any text, vagueness, or contradictory language.

Table 2 presents the frequency of each theme along with representative user quotes. To further protect user anonymity, all representative quotes were paraphrased into generalized summaries. These excerpts preserve the thematic essence of user experiences without directly reproducing identifiable language from individual reviews.

**Table 2:** Thematic Breakdown of App Store Reviews With Representative User Summaries Thematic analysis of user reviews for seven FDA-cleared PDTs, derived using non-negative matrix factorization and manual review. Themes represent recurring experiential dimensions such as engagement, access barriers, or technical issues. Each theme is presented with its frequency of occurrence and a representative, paraphrased user summary illustrating its core content. Quotes were generalized to protect user anonymity. This is an author-generated table.

Theme	Review Count (%)	Representative User Summary
Pediatric Benefit and Engagement	108 (43.7%)	Many caregivers reported that their children were engaged and showed signs of improved focus, though some mentioned that the app became repetitive over time.
Rewards and Incentives	32 (13.0%)	Users appreciated gamified elements like rewards or gift cards for increasing motivation, though a few questioned their clinical relevance.
Access and Activation Barriers	20 (8.1%)	Several users described difficulties accessing the app, citing issues with activation codes, long waitlists, or lack of support responsiveness.
Technical Issues and Updates	18 (7.3%)	Users frequently encountered technical problems such as crashes, login errors, or outdated versions that interfered with app functionality.
Emotional Reactions and Cost Sensitivity	15 (6.1%)	Some users expressed strong negative emotions or questioned the value of the PDTs, citing limited control options or lack of expected features.
Boredom and Frustration	9 (3.6%)	A subset of users felt the content lacked variety or accessibility features, leading to disengagement or frustration over time.

Cross-theme observations

While sentiment and theme were correlated in many cases (e.g., technical issues often co-occurred with negative ratings), this was not universally true. Some users left five-star reviews despite describing access frustrations, while others left one-star ratings but praised specific content elements. This underscores the complexity of interpreting digital therapeutic experiences through star ratings alone and highlights the value of full-text analysis.

EndeavorRx reviews were disproportionately represented in both volume and emotional intensity. Many reflected polarized parental responses, with some highlighting transformative improvements in attention and others dismissing the intervention as ineffective or difficult to use. Rejoyn, a more recent entrant, elicited both strong praise and concern, particularly around expectations and real-world outcomes.

## Discussion

This study demonstrates that publicly available app store reviews can offer not just RWE but real-world insights into how patients and caregivers interact with PDTs in daily life. Among 13 FDA-cleared products identified, eight had accessible reviews, and although sentiment skewed negative overall, thematic analysis revealed rich, multidimensional feedback that transcended simple star ratings. PDTs, despite their clinical rigor, operate within the same engagement landscape as consumer software, where friction, trust, and perceived value profoundly shape outcomes [8].

The most prevalent theme, "Pediatric Benefit and Engagement," reflected the promise and pitfalls of gamified interventions in children with attention-deficit/hyperactivity disorder (ADHD). While many parents praised improvements in focus and daily functioning, others described gameplay as repetitive or unsustained. One reviewer wrote, “This game actually helped my daughter focus. She looks forward to playing it-but sometimes gets bored with the same levels.” These dual sentiments echo a larger truth in digital medicine: therapeutic efficacy alone cannot guarantee sustained use or satisfaction.

"Rewards and Incentives" emerged as both a strength and a sticking point. For some users, badges and in-app milestones enhanced motivation. For others, they appeared disconnected from therapeutic goals or overly juvenile. This highlights the need for reward systems rooted not just in engagement [9], but in behavior-change theory tailored to the condition being treated.

Across several PDTs, "Access and Activation Barriers" remained a prominent source of user frustration. From waitlists and code-based entry systems to unclear prescription flows, reviewers frequently reported difficulties initiating or maintaining access. One user noted, “I’ve been prescribed this by my doctor but can’t get past the login screen. No one replies to support.” These bottlenecks are particularly problematic for populations with cognitive or mental health conditions, precisely those who may be least equipped to navigate them.

"Technical Issues and Updates" also featured prominently, with reports of app crashes, freezes, and failed updates. While some level of digital friction is expected in mobile applications, repeated functional breakdowns may erode user trust, especially when tied to health outcomes. These real-world usability challenges are often invisible in tightly controlled clinical trials but emerge forcefully in public reviews. Usability challenges, especially in mental health apps, have been repeatedly linked to poor adherence and early dropout [10]. Ensuring seamless onboarding and minimal digital friction is essential for clinical adoption [11].

Importantly, not all sentiment was negative. Rejoyn, a recently cleared PDT for major depressive disorder, received several glowing reviews. One user described it as “surprisingly helpful, almost like having a therapist in your pocket.” Another noted, “It helped me recognize patterns in my thinking that I hadn’t noticed before. I was skeptical, but it’s been a positive part of my treatment.” These endorsements speak to the potential of well-designed digital therapeutics to deliver perceived and meaningful benefits when user expectations, clinical need, and product design align. They also suggest that even in a field with many early challenges, there are success stories where patients credit the PDT with improving their health or well-being. Such positive cases are valuable for understanding what factors (e.g., interface, content, support) contribute to a satisfying user experience. Perceived benefit is a critical determinant of adherence to digital interventions and has been shown to influence clinical outcomes even in non-randomized settings [12].

However, the continuity of real-world review data remains vulnerable. Several products in this analysis underwent corporate transitions: reSET and reSET-O shifted from Pear Therapeutics to new ownership under PursueCare in 2023, Somryst was acquired from Pear by Nox Health, and AspyreRx moved from its original developer, Better Therapeutics, to Click Therapeutics during the study period. These handovers often reset app listings or migrated platforms, resulting in the loss of earlier user feedback. This creates a fragmented RWE landscape where valuable insights from prior users may be permanently inaccessible. If RWE is to inform real-world impact, it must be longitudinal, portable, and resilient to organizational change [13].

Limitations

This study has several limitations. It employs a mixed-methods design to explore both quantitative trends in sentiment and qualitative themes in user experience. While this dual approach enriches insight, the study remains exploratory and should not be interpreted as offering definitive comparative conclusions. App store reviews reflect voluntary, often emotionally charged responses and may not represent the full population of PDT users. Those who choose to leave reviews might differ systematically from silent users (for example, more dissatisfied individuals may be overrepresented). Sentiment classification was based solely on star ratings and may overlook sarcasm or instances where written feedback conflicts with the numeric rating. The thematic extraction, while conducted using both machine learning and manual review, may not capture all context-specific nuances, and the coding process could introduce subjective bias despite efforts at calibration.

Additionally, the use of publicly available product names, while potentially introducing perceived bias, was intentional to ensure transparency and clinical relevance. We emphasize that the inclusion of product-specific results does not imply endorsement and that all user reviews were paraphrased to avoid reidentification. Reproducibility may also be limited by the dynamic nature of app listings and review availability over time. Finally, as manual theme coding was performed by a single author, inter-rater reliability was not calculated, representing a potential source of interpretive bias.

We also note that several FDA-cleared PDTs lacked public reviews altogether, limiting the generalizability of insights predominantly to the subset of products with user engagement on open platforms. The relatively small dataset of 247 reviews, spanning seven PDTs, also limits the statistical generalizability of the findings. Disaggregation by app or sentiment further reduces sample size in each subgroup, increasing the risk of sampling error. Further, this introduces potential selection bias, as the included products may differ systematically from those without reviews in terms of user base, launch timeline, or marketing visibility. These factors may skew findings toward more accessible or higher-traffic apps. As such, the results should be interpreted as exploratory, hypothesis-generating insights rather than definitive conclusions about each individual product.

Finally, the dynamic nature of app marketplaces (with frequent updates and changing user bases) means our analysis provides a snapshot that could shift over time.

Implications and future directions

Despite its limitations, this study affirms that app store reviews offer a rich, unfiltered lens into the lived experience of digital therapeutics. They illuminate the gap between regulatory clearance and real-world adoption, between statistical efficacy and perceived value. While traditional clinical trials remain essential for regulatory approval, post-market user reviews may be uniquely positioned to surface issues related to access, usability, and trust -- domains often underrepresented in formal evidence generation. For developers, these reviews function as a real-time feedback loop, highlighting areas for iteration and improvement. For regulators and payers, they offer a scalable model for continuous surveillance of PDT performance. For patients and caregivers, they may serve as the most relatable form of insight, delivered not in p-values but in plain language and everyday frustrations or endorsements.

Looking forward, NLP and large language models (LLMs) present powerful tools to extract deeper insight from public review data [14]. Beyond basic sentiment classification, future work may leverage transformer-based models to detect nuance, sarcasm, and contextual meaning in unstructured feedback. With fine-tuning on domain-specific corpora, LLMs could automatically map reviews to existing technology acceptance and user burden frameworks, enabling standardized interpretation across products. Longitudinal analyses could track sentiment trends over time, especially in response to product updates, ownership transitions, or regulatory events. Additionally, future methods could integrate multimodal signals, including in-app behavior, chatbot transcripts, or biometric data, to contextualize review content within broader patterns of therapeutic engagement.

There is also a regulatory opportunity to formalize digital post-marketing surveillance using patient-generated content. This could take the form of optional, structured in-app feedback modules or integration with digital formularies that include patient satisfaction metrics alongside traditional coverage criteria. As PDTs become more embedded in clinical care pathways, stakeholder frameworks must evolve to capture not only outcomes but also experience.

Above all, the field must elevate user experience from a secondary consideration to a core pillar of digital therapeutic design, evaluation, and reimbursement. Real-world insight is no longer a luxury, it is an essential component of accountable, equitable, and scalable digital care.

## Conclusions

App store reviews of FDA-cleared PDTs provide a unique and largely untapped source of real-world insight into patient and caregiver experiences. While overall sentiment in this study skewed negative, a more detailed thematic analysis revealed substantial nuance, including meaningful reports of benefit, particularly in pediatric ADHD and major depressive disorder. Reviewers frequently highlighted barriers to access, technical challenges, and engagement limitations, underscoring the critical role of usability in therapeutic success.

These findings suggest that app-based reviews can serve as a form of digital post-marketing surveillance, capturing dimensions of value, trust, and frustration that traditional clinical trials may overlook. However, fragmentation of feedback due to corporate transitions and listing resets threatens the continuity of this evidence stream. As the field of digital therapeutics advances, greater attention must be paid to preserving the user voice across the full product lifecycle.

Ultimately, integrating qualitative user feedback into regulatory, reimbursement, and development frameworks may not only improve product design and adoption but also ensure that RWE delivers on its promise to reflect the realities of care.
